# Profiling alumni of a Brazilian public dental school

**DOI:** 10.1186/1478-4491-8-20

**Published:** 2010-08-18

**Authors:** Maria F Nunes, Erica T Silva, Laura B Santos, Maria G Queiroz, Cláudio R Leles

**Affiliations:** 1 School of Dentistry, Federal University of Goias, Goiania, Brazil

## Abstract

**Background:**

Follow-up studies of former students are an efficient way to organize the entire process of professional training and curriculum evaluation. The aim of this study was to identify professional profile subgroups based on job-related variables in a sample of former students of a Brazilian public dental school.

**Methods:**

A web-based password-protected questionnaire was sent to 633 registered dentists who graduated from the Federal University of Goias between 1988 and 2007. Job-related information was retrieved from 14 closed questions, on subjects such as gender, occupational routine, training, profits, income status, and self-perception of professional career, generating an automatic database for analysis. The two-step cluster method was used for dividing dentists into groups on the basis of minimal within-group and maximal between-group variation, using job-related variables to represent attributes upon which the clustering was based.

**Results:**

There were 322 respondents (50.9%), predominantly female (64.9%) and the mean age was 34 years (SD = 6.0). The automatic selection of an optimal number of clusters included 289 cases (89.8%) in 3 natural clusters. Clusters 1, 2 and 3 included 52.2%, 30.8% and 17.0% of the sample respectively. Interpretation of within-group rank of variable importance for cluster segmentation resulted in the following characterization of clusters: Cluster 1 - specialist dentists with higher profits and positive views of the profession; Cluster 2 - general dental practitioners in small cities; Cluster 3 - underpaid and less motivated dentists with negative views of the profession. Male dentists were predominant in cluster 1 and females in cluster 3. One-way Anova showed that age and time since graduation were significantly lower in Cluster 2 (*P *< 0.001). Alternative solutions with 4 and 5 clusters revealed specific discrimination of Cluster 1 by gender and dental education professionals.

**Conclusions:**

Cluster analysis was a valuable method for identifying natural grouping with relatively homogeneous cases, providing potentially meaningful information for professional orientation in dentistry in a variety of professional situations and environments.

## Introduction

Identifying professional profiles in follow-up studies of former students is an efficient way to organize the entire process of professional training and curriculum evaluation of an educational institution. Therefore, universities should continually revise the profiles of the professions for which they offer training.

Dental education may be planned to match societal demands and curriculum guidelines should address these regional needs. The dental profession in Brazil was especially influenced by changes in epidemiological traits of caries, growing demand for dental assistance, the reformulation of the public health care system and overall socioeconomic and cultural changes in recent years. These trends have occurred mainly in large cities, but inequalities in disease prevalence and access to dental care are still remarkable [1-3], despite the fast-growing addition of newcomers to the profession in Brazil.

Recent studies underlined recommendations for a strategic national oral health care plan for countries with both developed [4] and emerging economies [5]. The recent Brazilian national curriculum guidelines for university dental courses are consistent with public health policies, which emphasize the need for general dental practitioners focused on primary oral health care, with the ability to cooperate across different professional disciplines.

In Brazil, dental care assistance is provided in two ways: (1) a public health system focused on primary health attention, and (2) private dental care based on professional cooperatives and dental insurance companies, or fee-for-service health care. Both have serious shortcomings. Availability and accessibility are historical problems that affect the quality of public health services, owing to the high demand and the growing need of high complexity treatments. Private dental care is affected by cost and supplier factors. Treatment fees have great impact on access to dental care, and the utilization of dental services and supplier-induced demand - i.e. overconsumption of services generated by the economic self-interest of dental professionals - are common barriers to the need-demand-utilization process [6-8].

In this complex scenario experienced by dental care assistance and reformulation of Brazilian universities' curriculum, information about dental professionals' characteristics is lacking, including their practice context and personal views of the profession. The recognition of these factors provides strategic information for planning labor and educational policies. Thus, the aim of this study was to identify professional profile subgroups based on job-related variables, combined with their perception of professional practice in a sample of former students from a Brazilian public dental school.

## Methods

A cross-sectional study was planned to include former students of the School of Dentistry of the Federal University of Goias, who graduated in the period between 1988 and 2007. Academic and professional data was obtained from the University Registrar's Office and the Federal Council of Dentistry, respectively. The research project had been previously examined and approved by the Local Ethical Committee.

A web-based password-protected questionnaire regarding job-related variables and perceptions on profession was sent individually by e-mail to the former students using a software manager (SGAD, Cenatech, Goiania, Brazil). As the respondent accessed the e-mail message, a link with a numeric code redirected the respondent to a webpage on informed consent and acceptance for participation. The software manager allowed concurrent online monitoring of respondents' status throughout the process. In order to improve the response rate, the questionnaire was sent again two weeks later. One telephone reminder was performed one month after the first questionnaire.

The questionnaire consisted of 14 closed questions, including occupational routine, training, professional profits, income status, and self-perception of professional career. Questions emerged from discussions among the authors, reviewed by five experienced researchers who work with human resources in dentistry and tested in a group of 10 dentists who did not participated in the study sample.

From 1188 eligible former students, 546 were excluded from the sample because of: their failure to provide professional records, home address, telephone number or e-mail address (n = 367); cancellation or nonexistence of professional register (n = 174); or death (n = 5). The questionnaire was sent to the remaining 642 subjects, corresponding to 54.0% of the former students' population.

Descriptive statistics were obtained for nominal (frequency and percentage) and numerical (mean and standard deviation) data. The non-hierarchical two-step cluster analysis was used to divide samples into *n *number of clusters based on gender, and job-related and professional perception variables (14-item questionnaire), using an auto-clustering algorithm.

Alternative solutions with a different number of clusters were tried to disclose natural groupings other than the default auto-clustering option of the software. All proposed clustering solutions were selected according to interpretability and plausibility. Cluster analysis was used as an exploratory data analysis technique to reveal natural grouping from latent patterns in a large data set on the basis of a minimal within-group and a maximal between-group variation, without prejudgment. There are three stages to cluster analysis; partitioning/similarity (what defines the groups); interpretation of clusters (how to use groups); and profiling the characteristics of similar/partitioned groups (what explains the groups).

The two-step algorithm analysis allows subjects to be divided into an optimal number of clusters according to continuous and categorical variables. The variable importance for cluster segmentation was ranked by a Chi-square test in which each cluster group was tested against the overall group. Since multiple tests were performed, Bonferroni adjustments were applied to control the false-positive error rate. An alternative importance measure, which has the advantage of placing both types of variables on the same scale, is based on statistical significance values using -log^10 ^of the statistical significance (-log10 *P*-value). This transformation stretches the original scale from 0 to infinity (instead of a small band from 0 to 1), so that larger values of -log10 of *P*-value equate to greater significance.

One-way Anova followed by the Tukey post-hoc test were used to test differences among clusters according to three numerical variables: time since graduation; present age in years; and overall academic performance during degree.

The database of answers was exported to a data file of SPSS 16.0 software, which was used for clustering and all descriptive and hypothesis testing analyses.

## Results

The response rate was 50.9% (n = 322), 43.2% of them graduated before the year 1998 (1988-1997 group) and 56.8% graduated after 1997 (1998-2007 group). Respondents were predominantly female (64.9%), working in Goiania, the capital of the State of Goias (76.7%), and had an undergraduate degree as their highest professional training level (58.4%). Their age ranged from 23 to 49 years (mean = 34; SD = 6). No differences in gender (*P *= 0.218) and job localization (*P *= 0.778) were observed between the 1988-1997 and the 1998-2007 groups. On the other hand, the 1998-2007 group had significantly more professionals with an undergraduate degree only when compared to the 1988-1997 group (36.7 versus 74.9%; *P *< 0.001), as well as a lower age and time since graduation (*P *< 0.001).

By comparing the values of model-choice criteria across different clustering solutions and automatically determining the optimal number of clusters, the two-step exploratory cluster analysis revealed natural groupings of three separate groups with 52.2% (n = 151), 30.8% (n = 89) and 17.0% (n = 49) of the respondents (clusters 1, 2 and 3) respectively. The auto-clustering algorithm combined 289 cases (89.8%) in this three-cluster solution and 33 (10.2%) were excluded or unclassified.

Answers to the questionnaire are detailed in Tables 1 and 2, for job-related variables and perception about profession respectively. All variables used for clustering had a significant association with frequency distribution among groups (p < 0.001), except for the variables *type of health care *insurance and reported *job-related health problems *(Table 1). The relative importance of significant variables for the difference of each cluster is shown in Table 3, where within-group rank of variable importance for cluster segmentation is depicted for each cluster. The variables, which were significant for the cluster formation, were ordered individually for each cluster and the importance measures of each variable are expressed in Table 3 in the form of the frequencies of each category of the variable, and the Chi-square test and significance level (-log10 *P*-value and *P*-value). The greater the -log10 *P*-value, the greater the significance of the variable for the cluster formation. In each cluster, significant variables are in descending order of relevance for the clustering process, based on statistical significance.

**Table 1:** Distribution of cases according to job-related variables and gender in the 3-cluster solution

			Clusters (%)			
				
Variables	Categories	n (%)	1	2	3	P*
Considers dentistry as main professional occupation	Yes	291(90.4)	98.7	94.4	73.5	<0.001
	No/don't know	31 (9.6)	1.3	5.6	26.5	
						
Dental practice environment	Public/Private	59 (18.3)	11.9	24.7	24.5	0.019
	Private	134 (41.6)	45.0	48.3	34.7	
	Public/Privatized/Private	111 (34.5)	43.0	27.0	40.8	
						
Main professional activity	General dental care	131 (40.7)	11.3	93.3	44.9	<0.001
	Specialized dental care	140 (43.5)	74.8	3.4	34.7	
	Academic/administrative	51 (15.8)	13.9	3.4	20.4	
						
Main location of dental practice	Large city	254 (78.9)	90.7	53.9	91.8	<0.001
	Medium city	36 (11.2)	7.9	21.3	6.1	
	Small city	25 (7.8)	1.3	24.7	2.0	
						
Ordinary weekly workload	≤ 20 hours	39 (12.1)	5.3	1.1	49.0	<0.001
	≥21 and <40 hours	138 (42.9)	40.4	46.1	51.0	
	≥40 hours	139 (43.2)	54.3	52.8	0	
						
Highest qualification level	Undergraduate degree	94 (29.2)	1.3	74.2	24.5	<0.001
	Specialization degree	164 (50.9)	68.9	23.6	61.2	
	MSC and/or PhD	63 (19.6)	29.8	2.2	14.3	
						
Dentistry as the main source of income	Yes	286 (88.8)	97.4	94.4	69.4	<0.001
	No	35(10.9)	2.6	5.6	30.6	
						
Main family breadwinner	Yes	114 (35.4)	51.0	27.0	4.1	<0.001
	No	204 (63.4)	49.0	73.0	95.9	
						
Has or has had health problems which hinder dental practice	Yes	74 (23.0)	22.5	21.3	28.6	0.605
	No	248 (77.0)	77.5	78.7	71.4	
						
Gender	Female	209 (64.9)	53.0	71.9	89.8	<0.001
	Male	113 (35.1)	47.0	28.1	10.2	

*Chi-square test

**Table 2:** Distribution of cases according to perceptions of profession in the 3-cluster solution

			Clusters (%)			
				
Variables	Categories	n (%)	1	2	3	P*
Consider dentistry stressful	Very stressful	114 (35.4)	39.1	14.6	59.2	<0.001
	Somewhat stressful	166 (51.6)	48.3	76.4	30.6	
	Not stressful	42 (13.0)	12.6	9.0	10.2	
						
Satisfied with dentistry	Completely satisfied	89 (27.6)	39.1	19.1	4.1	<0.001
	Partially satisfied	181 (56.2)	60.3	71.9	36.7	
	Dissatisfied	51 (15.8)	0.7	9.0	59.2	
						
Would take dentistry again	Certainly or probably yes	155 (48.1)	57.0	56.2	10.2	<0.001
	Don't know	59 (15.5)	15.9	20.2	12.2	
	Probably or certainly not	117 (36.3)	27.2	23.6	77.6	
						
Self-rated professional success	Higher	235 (73.0)	93.4	67.4	28.6	<0.001
	Don't know	29 (9.0)	4.0	15.7	12.2	
	Lower	58(18.0)	2.6	16.9	59.2	
						
Self-rated professional performance	Higher	301 (93.5)	99.3	95.5	85.7	<0.001
	Lower	16 (5.7)	0.7	4.5	14.3	

* Chi-square test

**Table 3:** Relative importance of variables with statistical significance in the formation of clusters

Cluster	Variable	%	Chi- square	DF	**-log**_**10**_ *P*-Value*	*P*
1	Specialized dental care	85.0	62,1	2	13.5	<0.001
	Specialization degree	82.5	54,4	2	11.8	<0.001
	Professional success (yes)	65.6	29,3	2	6.4	<0.001
	Professional satisfaction (yes)	75.6	26,1	2	5.7	<0.001
	Main breadwinner (yes)	74.8	15,5	1	4.1	<0.001
	Large cities	59.6	13,6	2	2.9	<0.001
	Gender (male)	70.3	9,7	1	2.7	<0.001
						
2	Graduate degree	83.3	97,3	2	21.1	<0.001
	General dental care	68.0	95,4	2	20.7	<0.001
	Small and medium cities	88.0	40,9	2	8.9	<0.001
						
3	Professional satisfaction (no)	76.3	92,9	2	20.2	<0.001
	Weekly workload ≤20 hours	72.7	83,0	2	18.0	<0.001
	Professional success (no)	60.4	67,9	2	14.7	<0.001
	Would take dentistry again (no)	38.0	41,6	2	9.0	<0.001
	Dentistry as main source of income (no)	62.5	32,0	1	7.8	<0.001
	Main family breadwinner (no)	25.3	29,3	1	7.2	<0.001
	Dentistry as main occupation (no)	65.0	21,3	1	6.4	<0.001
	Gender (female)	23.4	13,2	1	3.6	<0.001
	Lower self-rated professional performance	58.3	12,6	1	3.4	<0.001
	Consider dentistry very stressful	28.7	13,2	2	2.9	<0.001

* - Log _10 _(Probability): greater value is more significant.

The interpretation of within-group rank of variable importance for cluster segmentation makes possible the individual characterization of clusters, as follows: Cluster 1, *specialist dentists *with higher *profits *and positive *views of the profession*; Cluster 2, *general dental practitioners *in small *cities*; Cluster 3, *underpaid *and less *motivated dentists *with negative *views of the profession. Male dentists *were predominant in cluster 1 and *females *in cluster 3.

A detailed description of clusters indicates that Cluster 1 basically contains predominantly *male dentists*, who are *specialists *and *practice specialized *oral health care for *most of their work time*. They work in *large municipalities*, are the *principal family breadwinners*, consider *themselves successful *and are *satisfied with their profession*. Cluster 2 is predominantly made up of *females*, with a *lighter weekly workload. Dentistry is not their main profession *or *source of income *and they are not the main *family breadwinners*. Negative aspects such as *stress, low professional self-esteem, dissatisfaction *and *feelings of regret *are present. Cluster 3 is made up of those who only have *a graduate degree*, work mainly in *general practice*, in small and medium-sized *municipalities *and are under low levels of *professional stress*.

**Figure 1:**
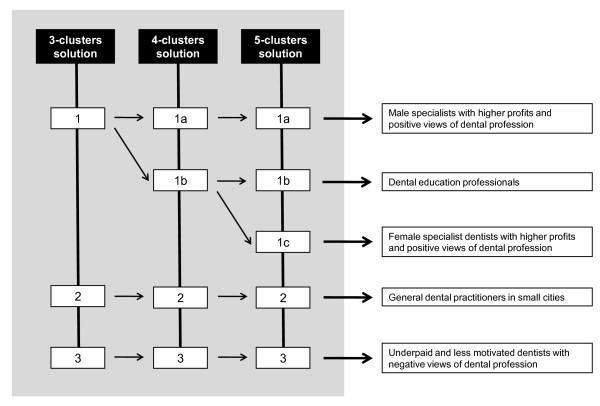
summary of all clustering solutions and group characterization

Alternative solutions with Clusters 4 and 5 showed specific discrimination of cluster 1 by *gender *and *dental education professionals*. Consequently, Cluster 1 was divided into two or three other subgroups: 1a/1c, gender-related subgroups of *specialist dentists *with higher *profits *and a positive *views of the dental profession*; and 1b, *dental education professionals*. The number of excluded cases was the same for the 4 and 5 cluster solutions. Figure 1 summarizes all clustering solutions and group characterization.

Between-group comparison of clusters according to numerical variables (Table 4) showed that *age *and *time since graduation *were significantly lower in Cluster 2 (*P *< 0.001). There was a significant difference for the lower values of Cluster 1a (4 and 5-clusters solutions) for *academic performance *in undergraduate courses.

**Table 4:** Between-group comparison of cluster according to three numerical variables (time since graduation, age and overall academic performance in undergraduate courses).

Continuous variables	3-cluster solution		4-cluster solution		5-cluster solution	
	
	Cluster	Mean (SD)	Cluster	Mean (SD)	Cluster	Mean (SD)
Time since graduation (years)	1	11.07 (5.8)^A^	1a	11.96 (5.4)^A^	1a	12.04 (5.4)^A^
	3	9.98 (5.6)^A^	1b	10.02 (6.1)^A^	1b	10.18 (6.0)^A^
	**2**	**6.62 (5.8)**^**B**^	3	8.70 (5.7)^A^	3	9.98 (5.6)^A^
			**2**	**6.38 (5.7)**^**B**^	1c	9.94 (5.9)^A^
					**2**	**5.95 (5.6)**^**B**^

Present age (years)	1	35.06 (5.9)^A^	1a	36.05 (5.6)^A^	1a	35.96 (5.5)^A^
	3	34.14 (5.3)^A^	3	33.94 (5.5)^AB^	1b	34.39 (5.7)^A^
	**2**	**31.51 (6.4)**^**B**^	1b	33.83 (6.2)^AB^	3	34.12 (5.6)^AB^
			**2**	**31.40 (6.3)**^**B**^	1c	33.86 (6.4)^AB^
					**2**	**30.92 (6.3)**^**B**^

Overall academic performance (0-10 scale)	2	7.53 (1.0)^A^	2	7.48 (1.0)^A^	1b	7.61 (0.9)^A^
	3	7.42 (0.9)^A^	1b	7.47 (0.9)^AB^	2	7.47 (1.0)^AB^
	**1**	**7.22 (0.9)**^**A**^	3	7.39 (0.9)^AB^	1c	7.46 (0.8)^AB^
			**1a**	**7.06 (0.9)**^**B**^	3	7.33 (0.9)^AB^
					**1a**	**7.03 (0.9)**^**B**^

- Different letters indicate statistically different clusters (One-way Anova followed by Tukey's test); *P *< 0.05;

- Clusters with lower values are highlighted in bold - Cluster 2 were younger and have shorter time since graduation, and Cluster 1/1a had lower academic performance during degree course.

## Discussion

This study revealed natural groupings among former students of a Brazilian public university according to *job-related issues *and *perception about profession*. Diversity in professional profiles shows the dynamic nature of dentistry as a profession and reveals important underlying factors influencing dental careers. The skills, motivation and commitment of the health care workforce in general are increasingly recognized as being intimately linked with the performance of health systems, and thus important for research [9]. Recent technical advances, changes in the public and private health systems, an increasing number of professionals, increasing female enrollment in health professions, and changes in educational guidelines are major challenges facing dentistry today in Brazil.

Previous studies aimed at identifying dentists' professional profiles from different perspectives [4,10,11]. Gender-related studies observed that women are more inclined to have a lower weekly workload owing to family commitments [10,11]. Nunes and Freire [12] studied quality of life profiles in Brazilian public health dentists and reported a low quality of life in physical and psychological domains and a high quality of life in social relationships and environmental domains, which were associated to self-rated quality of life, current health status and job satisfaction.

Our data was collected using a web-based questionnaire builder and analyzer, which can provide functions for researchers to create questionnaires in a fast and easy manner, and increase the response time and rate. However, the link to the questionnaire was provided by e-mail and, consequently, failure to locate former students and identify a valid e-mail address significantly reduced the number of eligible subjects from the final sample. Almost half of the sent questionnaires were unanswered, most of them probably due to a failure to access an e-mail account. This is definitely a major problem with web-based questionnaires, since it is estimated that only 34.4% of the Brazilian population are internet users and only 3.5% are broadband subscribers [13]. The telephone contact was also tried as a strategy to increase response rate, however the respondents return was insignificant. These findings reveal the difficulty of the Council of Dentistry to update the addresses, e-mail and phone numbers of dentists. Non-response bias needs to be considered, although non-response rates were distributed similarly among the different sample groups.

Cluster analysis is a relatively uncommon method used in dental research, although commonly used for market segmentation purposes. To summarize, cluster analysis is a way of grouping cases of data based on the similarity of responses to several variables, and is useful mainly in situations where there are hundreds of people and lots of variables, which would become very cumbersome and almost impossible to interpret. However, there some things to be aware of when conducting cluster analysis, mainly because solutions cannot be unique, since they are based on algorithms rather than formal mathematics. Limitations of cluster analysis include the proper selection of method, since different methods of clustering usually give very different results, also the results will be affected by the way in which the variables are ordered and the analysis is not stable when cases are dropped. Running different alternative clustering solutions with careful interpretation of clusters and profiling characteristics, according to the study interest, is essential in determining the number of clusters in the final solutions, since no method for validation is available for an optimal solution. Considering that cluster analysis is very sensitive to the entry of new variables, we opted to perform clustering using the categorical variables separately, and subsequently performed between-group comparison of clusters with the numerical variables.

Descriptive analysis showed some universal characteristics of dentists' population elsewhere: predominance of *women*, high levels of professional involvement (*dentistry as main occupation *and high *weekly workload*), predominance of *workers in private dental service*, and a tendency toward *specialization *and *concentration in large cities *[4,5,10]. Bravo-Péres [1] found a similar situation in Spain in 2004 and Brown and Lazar [14] described how, in the United States of America, the decline in private practice started at the beginning of the 1990 s. In Brazil, a similar situation occurred at the same time when there was an increased demand and utilization of public dental services. Even though private practice still predominates, there is a decreased tendency, because of the number of professionals in the public sector as a result of public health policies. Similarly, the predominance of women among respondents is in conformity with the greater prevalence of female students in dentistry. Health professions have long been characterized by gender disparities, but some professions, such as dentistry, have historically been dominated by males. Over the past decades these disparities have narrowed or even reversed [14].

Only a minority considered *dentistry as a low-stress profession *(13.0%) and the majority reported they were in a *healthy state *with no health problem that could hinder their professional practice (77.0%). On the other hand, it's important to observe that almost a quarter of the sample (23.0%) reported have been unable to exercise their professional activities fully at some time during the previous six months. Of these, 68.9% said that their illness was totally or partly related, to their professional practice. Dentistry is recognized as a source of stress for professionals and is described frequently as a cause of many health problems [12,15-17].

Contrasts in *perceptions about the profession *were observed but, in general, positive views were more prevalent. *Job satisfaction *is considered to be a subjective variable which could differ in significance from one person to the next, and even for a certain person at different times. It can vary according to circumstances, work atmosphere and culture. Chambers [18] reported that half of dentists would not choose dentistry again if they had the opportunity. However, the number of those who abandon their profession voluntarily is lower than that of those in the overall population who change careers, by a ratio of 1 to 15. This apparent contradiction was confirmed in Brazil by Moimaz et al. [15], where the majority of women said they were satisfied, but more than 50% would not encourage their children to choose dentistry as a profession.

Clustering identified three major groups with other alternative partitions (4 and 5 cluster solutions). Cluster 1a was characterized as *male specialists *with higher *profits *and *positive views of the profession*. They were basically those who had *graduated earlier *and are undoubtedly better *established in the profession*. Shugars et al. [19] found similar characteristics among Californian dentists, where the most satisfied were the oldest, reporting higher incomes, were better qualified and worked with auxiliary personnel. In New Zealand in 2008, Ayers et al. [11] also concluded that males were more satisfied professionally than females. Conversely, in our study this group was found to have the lowest academic performance in undergraduate courses among all other groups.

Subsequent division of cluster 1 into 1b and 1c revealed *dental education professionals *and *female specialists*, who differ from cluster 1a in respect to *academic performance *(significantly higher in cluster 1b) and *gender*.

Cluster 2 comprised younger, more recently *graduated dentists *(Table 4), and consequently includes the majority of those who only have a *graduate degree *(74.2%) and *general practitioners *(93.3%). Another relevant fact is that professionals in this group work in small or medium-sized municipalities (46.0%), suggesting a tendency towards moving to a country town to exercise the profession, which occurs principally because of work opportunities. The positive views of the profession of this group were also observed by Baldwin et al. [20], who concluded that very young dentists tend to have a very positive attitude towards their work and career.

Cluster 3 was the least satisfied with dentistry, characterized predominantly by *females *(89.8%). The recent tendency towards the feminization of dentistry reinforces the need for a better investigation into this segment of the population to improve their quality of work life. Ayers et al. [11] investigated gender differences in the practice and satisfaction with dental careers and found that females were more dissatisfied with their careers, and that a large number of them would not choose dentistry again if they had the opportunity. Baldwin et al. [20] studied an English sample of recent graduates and reported that males were more self-confident in their professional practice and that females had a greater fear of litigation, and reported more experience of discrimination. In Brazil, Moimaz et al. [15] concluded that, although the majority reported satisfaction with the profession, the amount who reported financial and health problems, complaints and disappointment would suggest dissatisfaction, sometimes unconscious, of females in dental practice.

This group also contains a significant proportion of those who did not consider *dentistry as their main occupation *(26.5%), nor their *main source of income *(30.6%), and were not the *main family breadwinner *(95.9%). All these aspects denote discontent with the profession, corroborating the study of Moimaz et al. [15]. Other studies found that major causes of dissatisfaction with the dental profession were low income [19,21], the lack of personal time, intense competition and market saturation [15,19,22]. Profiling characteristics were defined as underpaid and less motivated dentists with negative views of the profession.

This study provides potentially meaningful evidence for the current context of curriculum reformulation in Brazil, and policies for educating and training dental professionals. It also gives useful information about the outcomes of the dental career of former students as an important tool for orientation of current students. Continuous assessments of these aspects are crucial to reaffirming patterns and identifying new trends, towards an understanding of the differences and similarities among professional profiles at different times, mainly after curriculum reformulation.

The differences among clusters reinforce the need for additional studies to investigate the dental career under different professional conditions, opportunities and environments. Gender difference in job satisfaction, for example, is an important aspect to be studied, especially in the current context of the increasing enrollment of women in the dental profession. Additionally, it is important to investigate reasons for the greater satisfaction among women engaged in teaching and administrative positions than those in clinical activities.

In our study, it was not possible to infer the causes of professional dissatisfaction. These questions need to be studied at greater depth and may result in the formulation of specific academic and professional policies at the local and national perspective. Our results certainly have remarkable relevance for the local and regional scenario, but other dental population may show different results since laws and regulations regarding education and health insurance vary considerably worldwide.

## Conclusions

The natural groupings identified in this cohort of Brazilian dentists reveal great diversity in professional profiles with respect to aspects of the dental career and satisfaction within the profession. Groups also presented differences in previous academic performance and time since graduation. Cluster analysis was a helpful method for identifying natural grouping with relatively homogeneous cases, providing potentially meaningful information for continuing professional development in dentistry and promotion of specific policies for human resources in oral health care. Findings suggest that understanding the underlying issues influencing dental careers is essential to retaining a motivated dental workforce in the Brazilian health system and to helping new entrants into the profession to have realistic and positive professional expectations.

## Competing interests

MFN, MGQ and CRL are academic staff at the School of Dentistry of the Federal University of Goias. CRL is the coordinator of the Postgraduate Program. ETS and LBS are graduate and undergraduate students, respectively, at the School of Dentistry of the Federal University of Goias.

## Authors' contributions

MFN, MGQ and CRL conceived and designed the study. CRL performed the statistical analysis and helped to draft the manuscript. MFN, ETS and LBS participated in the design of the study and helped to collect the data. All authors read and approved the final manuscript.
